# Cohort Profile: 46 years of follow-up of the Northern Finland Birth Cohort 1966 (NFBC1966)

**DOI:** 10.1093/ije/dyab109

**Published:** 2021-08-30

**Authors:** Tanja Nordström, Jouko Miettunen, Juha Auvinen, Leena Ala-Mursula, Sirkka Keinänen-Kiukaanniemi, Juha Veijola, Marjo-Riitta Järvelin, Sylvain Sebert, Minna Männikkö

**Affiliations:** 1 Northern Finland Birth Cohorts, Infrastructure for Population Studies, Faculty of Medicine, University of Oulu, Oulu, Finland; 2 Center for Life Course Health Research, Faculty of Medicine, University of Oulu, Oulu, Finland; 3 Medical Research Center Oulu, Oulu University Hospital and University of Oulu, Oulu, Finland; 4 Unit of Primary Care, Oulu University Hospital, Oulu, Finland; 5 Oulunkaari Health Center, Ii, Finland; 6 Healthcare and Social Services of Selänne, Pyhäjärvi, Finland; 7 Healthcare and Social Services of City of Oulu, Oulu, Finland; 8 Department of Psychiatry, Research Unit of Clinical Neuroscience, University of Oulu, Oulu, Finland; 9 Department of Psychiatry, University Hospital of Oulu, Oulu, Finland; 10 Department of Epidemiology and Biostatistics, School of Public Health, Imperial College London, London, UK; 11 MRC-PHE Centre for Environment and Health, School of Public Health, Imperial College London, London, UK; 12 Department of Life Sciences, College of Health and Life Sciences, Brunel University London, London, UK

Key Features

- The Northern Finland Birth Cohort 1966 (NFBC1966) was originally set up to identify a variety of maternal biological, behavioural and socioeconomic risk factors involved in preterm birth and intrauterine growth retardation, and to study the consequences of these early adverse outcomes on children’s subsequent morbidity.
- At baseline, all mothers with expected dates of delivery between 1 January and 31 December 1966 were recruited from the two northernmost provinces in Finland. The NFBC1966 study population comprised 12 231 children and their parents.
- The follow-ups have been conducted at four different time points, i.e*.* at 1, 14, 31 and 46 years of age. Participation rates in the latest follow-up were 69% for the questionnaires (*n* = 7146) and 57% for the clinical examination (*n* = 5832).
- Collected data include prenatal and early life measurements and information on motor, social, psychological and mental development in childhood. In adulthood, data on social background, lifestyle, medication, diagnosed diseases, organ-specific and psychiatric symptoms, workload and occupational health, economy, personal traits, functioning, quality of life, use of health services and family history of diseases.
- NFBC data are used widely in international collaboration projects.
- Permission to use the data for research purposes can be applied for via the electronic material request portal [https://www.oulu.fi/nfbc/].

## Why was the cohort set up?

The Northern Finland Birth Cohort 1966 (NFBC1966), originally named ‘Northern Finland Premature Birth Study’, was started in 1965 by Professor of Pediatrics Paula Rantakallio to respond to the prevalent problems of perinatal mortality and morbidity in relation to low birthweight.^1^^,^^2^ Specifically, the project was initiated to: (i) identify a variety of maternal biological, behavioural and socioeconomic risk factors involved in preterm birth and intrauterine growth retardation; and (ii) study the consequences of these early adverse outcomes on children’s subsequent morbidity.

This cohort profile describes the recruitment of the NFBC1966 and follow-up studies until the age of 46 years. The prospective data form a unique resource, located at the University of Oulu in Finland, which allows for studying the life course origins of health as well as the incidences and clustering of diseases and their genetic, biological, psychosocial and behavioural backgrounds. The follow-ups of the cohort participants will continue with University of Oulu support for the NFBC infrastructure.

## Who is in the cohort?

The NFBC1966 was set up in the two northernmost provinces of Finland, Oulu and Lapland, which together covered an area equivalent to 48% of Finnish territory and 13.2% of population in 1966, i.e. a selected area of 160 000 km^2^ with approximately 604 000 inhabitants (Statistics Finland: https://www.stat.fi). All mothers with expected dates of delivery between 1 January and 31 December 1966 were recruited. The data were collected at 157 antenatal clinics of the district by a trained staff of 188 local midwives. Mothers were recruited based on the calculated expected date of delivery at the first antenatal visit. The gestational age was determined either from the first day of the last menstrual period or from the expected due date estimated from the date of commencement of fetal movements and progress of the pregnancy.^1^ A total of 12 055 mothers were recruited in the study. In total, there were 12 068 deliveries (13 mothers had two deliveries during the recruitment period), which included the births of 12 231 children included in the cohort. Those born in 1966 (*n* = 11 979) comprised 95.6% of all children born in the area (*n* = 12 527 according to Statistics Finland). A small portion of the births occurred towards the end of 1965 (*n* = 189) and early 1967 (*n* = 63), i.e. children born before or after the expected date of birth. The study covered all live-born infants and stillborns (*n *= 173) with birthweight of 600 g or more. Finally, 12 058 live-born children were included in the follow-ups, i.e. 5890 girls and 6168 boys, of whom were 314 twins (see Table 2 under ‘Loss to follow-up’).

## How often have they been followed up and what has been measured?

Each follow-up study of the NFBC1966 has been evaluated by the regional ethical committee of the Northern Ostrobothnia Hospital District (EETTMK 94/11, 17.09.2012).

**Figure 1:**
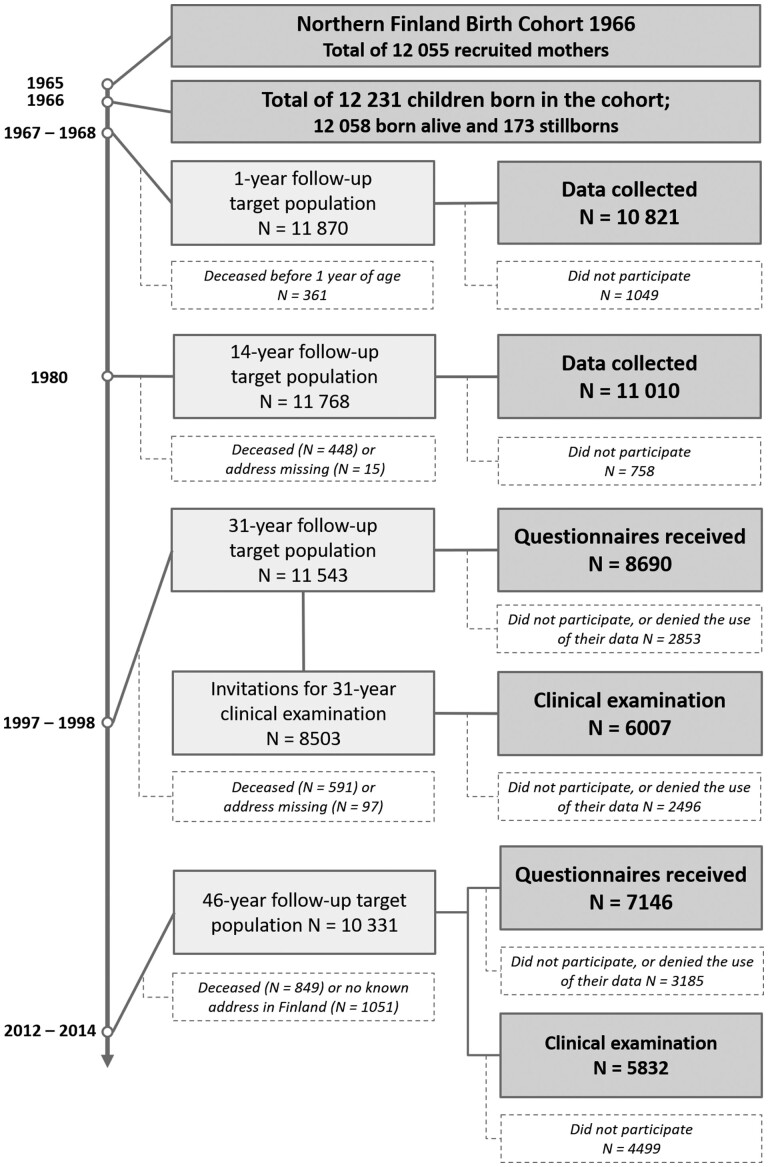
The flowchart of the Northern Finland Birth Cohort 1966 study. Target population at each time point consists of all original cohort members alive with a known address

**Figure 2:**
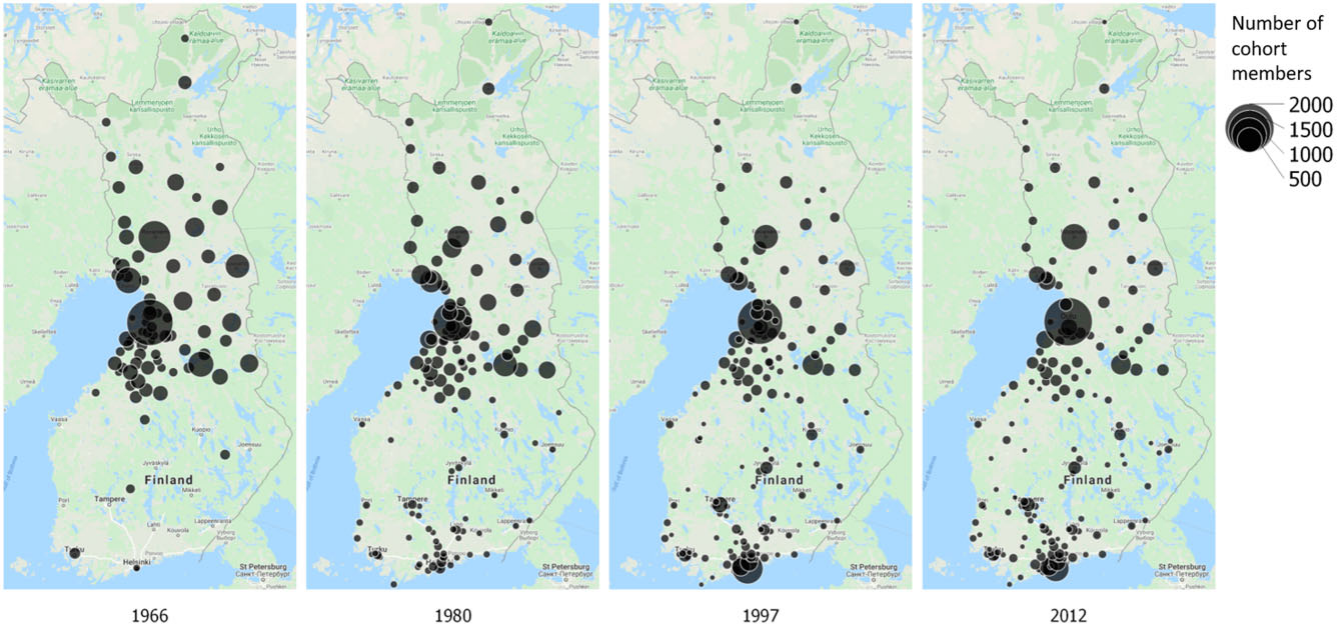
The proportional symbol map of the place of residence of Northern Finland Birth Cohort 1966 members in Finland at the time of birth (mother’s address) and 14-, 31-, and 46-year follow-ups. Places with five or less cohort members are not shown. Figure was created with QGIS 3.8 (QGIS Development Team, 2019, QGIS Geographic Information System, Open Source Geospatial Foundation: http://qgis.org)

The first set of data available for the NFBC1966 dates on average from the 16th gestational week (maternity card data transferred into cohort databases). Questionnaires on sociodemographic-, lifestyle- and disease-related factors were collected at antenatal clinics during the 24th to 28th gestational weeks (89.9% of the mothers) and, in cases of non-attendance during those weeks, were completed later in pregnancy or after delivery (10.1%).^1^ The follow-ups for the whole cohort have been conducted at four different time points i.e. at 1, 14, 31 and 46 years of age. The latest clinical follow-up was completed on 1 March 2014. Contact details were updated from the Finnish Population Register Centre (Digital and Population Data Services Agency since January 2020). Linkages with national register-based data provide further information on health between the follow-ups and are currently available until the age of 50 years. The follow-up and the attrition in the NFBC1966 are described in Figure 1 and later in the text. Since 1966, the cohort participants have migrated within the country especially towards southern Finland, and this provides an opportunity to study aspects of geography and health (Figure 2).

In the beginning, the research in NFBC1966 focused on low birthweight. Since then, multiple research areas have emerged in a wide range of health-, society- and demography-related categories. As a general overview, the first follow-ups during infancy and childhood until the age of 14 years covered research areas associated with motor, social, psychological and mental development and related aspects of health.^3^^,^^4^ Whereas the early follow-ups were only questionnaire based, the extensive follow-up studies in adulthood (at 31 and 46 years of age) included both questionnaires (web-based filling optional at 46 years) and clinical examinations. Parental factors and data collected at birth and in the follow-up studies are presented in Table 1 as well as on the cohort website [https://www.oulu.fi/nfbc]^5^ together with the detailed study protocols for clinical examinations. Prospects of each phase of the follow-up are briefly described below.

**Table 1:** The Northern Finland Birth Cohort 1966 data collections^a^

Parental factors	Birth	1 year	14 years
Mothers *n* = 12 055; fathers n = 11 068	Newborns n = 12 058 (live-born)	*n* = 10 821	*n* = 11 010
Parents’ age, occupation, education, marital status, economy (mothers, fathers) Parents’ place of residence, housing Parents’ history of cardiovascular diseases in a family Parents’ history of other diseases Wantedness of the pregnancy Birthweight of previous children Maternal: weight, height, miscarriages, weight gain during pregnancy, age at menarche, stillbirths, parity, blood pressure (highest reading in 2-4th and 5-9th month), smoking 1 year before pregnancy and during pregnancy (no. of cigarettes), working during pregnancy, workload, Hb in the 3rd, 7th and 9th gestational month and before birth, oedema, albuminuria, certain infections, depression and subjective well-being during pregnancy, medication with type and dosage	Birthweight Birth length Gestational age Placental weight and abnormalities, umbilical cord Mode of delivery Apgar score Congenital abnormalities Detailed information on neonatal hospital admission and diagnoses	Development (e.g. standing, walking, talking, neurological abnormalities) Weight (also at 6 months) Height (also at 6 months) Head circumference Vitamin supplementation Diseases, hospitalizations, outpatient visits (causes) Medication Vaccination	Weight, height, waist-hip circumference, leg length Physical activity Smoking, alcohol drinking, drugs Schooling Family structure Social mobility Parental occupation Long-term diseases (asthma, atopy, diabetes) Central nervous infections Brain damage Parental history of chronic diseases

31 years	46 years	31 years	46 years
Postal questionnaire *n* = 8690	Postal questionnaire *n* = 7146	Clinical examination *n *= 6007	Clinical examination *n* = 5832

Marital status, family structure Migration Education, occupation and working life, income Smoking, alcohol drinking, medication, nutrition Cardiovascular disease or high blood pressure (diagnosed by a physician) Long-term diseases, ISAAC questions about asthma, atopy and wheezing Age at menarche Birthweight of own children Information on women’s own pre-eclampsia and deliveries Family history of chronic diseases	Marital status, family structure Migration Education, occupation, working life, income, risk-taking Smoking, alcohol drinking, medication, nutrition Cardiovascular disease or high blood pressure (diagnosed by a physician) Long-term diseases, ISAAC questions about asthma, atopy and wheezing Life satisfaction, reserves of strength, sleep Pregnancy, endometriosis, pre-eclampsia, PCOS, infertility Cognitive tests (Paired Associates Learning, PAL)	Weight, height, waist-hip circumference Blood pressure, pulse (a mercury sphygmomanometer) Physical fitness (step test) Back muscle strength Compression force of hands (hand grip dynamometer) Skin allergy tests (prick) Spirometry (Vitalograph P-model spirometer) Bone density (DTX 200, Osteometer Meditech) (*n* = 1000)	Weight, height, waist-hip circumference, bioimpedance (InBody 720) Blood pressure (brachial), pulse (Omron M10) Physical fitness with 2-week accelerometry measurements (Polar RX800RC, Hookie AM20) Skin allergy tests (prick) Spirometry (MasterScreen Pneumo, Spira) 15-lead ECG (CAM14 GE Healthcare) Heart rate variability (Polar RX800RC. Polar WearLink belt), step-test, brachial blood pressure (in sitting and standing positions), baroreflex measurement and breathing frequency Back muscle strength Pressure pain threshold (algometer, Somedic), thermal perception thresholds and tolerance (modular sensory analyser, Somedic) Spinal and ankle movement tests, low back movement control tests (SpinalMouse Med Pro) Compression force of hands (hand grip dynamometer) Glucose tolerance test Eye examination (vision, refraction, intraocular pressure, thickness of cornea, field of vision, eye photography), randomized for half of the cohort^b^

31 years biological samples and analyses Maximum *n* = 6007	46 years biological samples and analyses Maximum *n* = 5832		46 years extended study protocol for Oulu area

Biological samples collected: plasma, serum, EDTA-blood (DNA extracted) Laboratory measurements such as blood count, high-sensitivity CRP, glucose, insulin, triglycerides, total LDL-, HDL-cholesterol, vitamins C and D GWAS (*n* = 5402) NMR metabolomics (*n* = 5739) Methylation (*n* = 807) Telomers (*n* = 1450)	Biological samples collected: plasma, serum, EDTA-blood (DNA extracted), PAXGene for RNA, cells, hair, saliva, faeces, urine Laboratory measurements such as blood count, high-sensitivity CRP, glucose, insulin, triglycerides, total LDL-, HDL-cholesterol, vitamin D, S-T4V, S-TSH, S-TPOAb, cortisol NMR metabolomics (*n* = 5800, OGGT *n *= 4900) Methylation (*n* = 760) Telomers (*n* = 5783) Immunoglobulins (*n* = 5774)		Echo and carotid ultrasound (Vivid E9, GE Healthcare) (*n* = 1156) Dermal examination by physician (*n *= 1930) X-ray of knee, ankle, foot, (Simax/Fuji FCR XG-1) and OPTG (Orthopantomoraph OP100) (*n* = 1975) Lumbar MRI (1.5 T imaging Signa HDxt, General Electric) (*n* = 1534) Oral health: dental caries, periodontitis, occlusion, temporomandibular disorders, facial structures (3 D-imaging of mouth and teeth, Cadent iTero3D) (*n* = 1964)

ISAAC, International Study of Asthma and Allergies in Childhood Questionnaires; PCOS, polycystic ovary syndrome; ECG, electrocardiogram; CRP, C-reactive protein; LDL, low-density lipoprotein; HDL, high-density lipoprotein; GWAS, genome-wide association study; NMR, nuclear magnetic resonance; EDTA, ethylenediaminetetraacetic acid; DNA, deoxyribonucleic acid; RNA, ribonucleic acid; S-T4V, serum tetraiodothyronine; S-TSH, thyroid-stimulating hormone; S-TPOAb, serum thyroid peroxidase antibodies; OGGT, oral glucose tolerance test; OPTG, orthopantomography.

a More detailed information about each follow-up is available at [https://www.oulu.fi/nfbc/nfbc1966_1986].

b Detailed study protocol has been described in Saarela *et al*. at [http://www.biomedcentral.com/1471-2415/13/51].

### 1-year follow-up

The children attended regular visits to the welfare clinics (on average 10 visits during the first year), where their developmental data were collected. At the 1-year child welfare clinic visit, which included doctor and public health nurse examinations, the research forms were filled in by the public health nurses. At this stage, 11 870 (98.4%) of the cohort members were alive. In 95% of the cases, information was collected by the age of at least 11.5 months (the mean age was 1 year and 10 days).

### 14-year follow-up

In 1980, when the cohort members were 14 years of age, a survey regarding their health and physical condition was conducted. Questionnaires were completed primarily by the teenagers themselves (*n* = 11 010). If they did not answer, a shorter questionnaire was sent to the parents (*n* = 389). Finally, information was collected from the schools (*n* = 354) if there was no response from the family. Of the whole cohort, 94% of children attended school at a level appropriate for their age (normal class or above), 3.8% attended a class below their age and 2.2% either attended a school specialized for children in needs or had been exempted from compulsory education.

### 31-year follow-up

The 31-year field study in 1997 focused on somatic and mental health and work ability in the NFBC1966. It included postal questionnaires and a clinical examination. The questionnaires were sent to all individuals with a known address in Finland (10 631) and abroad (695), and later for a group whose addresses were obtained later (215). The clinical examination was performed only among individuals living in Northern Finland (7209) and the Helsinki area (1284). In total, 75.3% answered the postal questionnaires and 70.7% of the target population participated in the clinical examination. In all, 5973 cohort members both filled the questionnaire and participated in the clinical examination.

The clinical examination was performed by trained professionals and included measurements such as anthropometric parameters, blood pressure, physical performance, skin allergy (prick test) and spirometry. In addition, overnight fasted blood samples were collected, and many hormones, cytokines and metabolic parameters have been analysed. White blood cell DNA was extracted, and genome-wide genotyping performed (Illumina Human CNV370-duo Bead Array).^6^

### 46-year follow-up

In 2012, at the age of 46 years, all cohort members who were alive and living in Finland with a known address (*n* = 10 331) were invited to participate in a large health examination that consisted of both questionnaires and clinical examination. The four questionnaires were named as: (i) Background, lifestyle and health survey; (ii) Economic, working life and resources survey; (iii) Opinions and experiences; and (iv) Supplementary questions. Together they included items about social background, lifestyle (sleep, smoking, physical activity and nutrition), medication, diagnosed diseases, organ-specific symptoms (musculoskeletal, gastrointestinal, ophthalmological, dental, respiratory, neurological, dermal and gynaecological symptoms), psychiatric symptoms (depression and anxiety), workload and occupational health, economy, personal traits, functioning, quality of life, use of health services and family history of diseases. Questionnaires were received from a total of 7146 cohort members. In all, 5055 subjects (70.7%) answered all four questionnaires, with variation from 5643 to 6834 between different parts (Figure 1).

Basic clinical health examinations were offered by post to all cohort members and performed by three research nurse teams in 36 towns all over Finland (*n* = 5832). Examinations comprised, for example, anthropometric parameters, brachial blood pressure, physical performance, 15-lead electrocardiogram, heart rate variability test, pressure pain threshold and tolerance test (Somedic AB, Hörby, Sweden), skin allergy test (prick test), spirometry, oral glucose tolerance test (0-, 30-, 60- and 120-min samples), cognitive test (Paired Associative Learning test, PAL, Cambridge Cognition, Cambridge, UK) and objective measures of physical activity and sleep through a 2-week accelerometer measurement using the wrist with Polar Active (Polar Electro Oy, Kempele, Finland) and using the hip with Hookie AM20 (Traxmeet Ltd, Espoo, Finland) with a diary. In addition, a randomized sample of 3070 subjects attended ophthalmological examination.

A more detailed clinical examination was performed for a subpopulation living in the area of Oulu (Table 1). This included thermal perception threshold and tolerance tests, detailed dental examinations with dental 3 D scanning and orthophantomogram (OPTG), central blood pressure, autonomic nervous system tests, heart (echo) and carotic artery ultrasound, radiography of knees, ankles and feet, spinal and ankle movement tests, lower back movement control impairment tests and sciatica-neurology tests. In addition, lumbar magnetic resonance imaging (MRI) was performed for 1534 subjects.

Biological samples (blood, feces, urine, saliva and hair) were obtained from all who attended clinical examination. DNA has been extracted from samples collected at 46 years of age enabling, for example, the analyses of repeated measures of DNA methylation and telomers at 31 and 46 years of age in relation to changes in health/disease.

At 46-year follow-up, of those who participated in the clinical examination, the majority (*n* =  5822) answered at least one of the questionnaires. A total of 4016 answered both 31-year and 46-year questionnaires (at least one) and participated in both clinical examinations.

### Subpopulation follow-up studies

Several small follow-ups have emerged from the large follow-up studies performed in NFBC1966. These have been conducted to get more detailed information in relation to specific issues, for example neurological diseases or ophthalmological and hearing-related defects. These subpopulation studies are described in the Supplementary Table S1, available as Supplementary data at *IJE* online.

### Linkage to national registers

Using Finnish individual social security numbers, the data from NFBC1966 follow-ups have been linked with information obtained from various Finnish national registers, such as the Population Register Centre, Finnish Centre for Pensions, Statistics Finland, Social Insurance Institution of Finland, Finnish Institute for Health and Welfare and National Board of Education.

### Loss of follow-up and missingness

The participation in different follow-ups has ranged 69% and 94% regarding questionnaires and 57% to 71% regarding clinical examinations. The attrition analyses were conducted using variables from the first questionnaire and from registers. Information about family socioeconomic status and mother’s education and area of living was obtained at the baseline of the cohort. Information about cohort members’ socioeconomic status (SES), employment, education, marital status and their own offspring was obtained from Statistics Finland. More detailed information on the attrition analyses is presented in Table 2.

**Table 2:** Attrition analyses of the Northern Finland Birth Cohort 1966 follow-ups

	1-year follow-up					14-year follow-up				
	Participation		No participation^a^			Participation		No participation		
	*n*	(%)	*n*	(%)	*P*-value	*n*	(%)	*n*	(%)	*P*-value
Questionnaire										
Gender										
Male	5502	(90.3)	592	(9.7)	0.034	5555	(92.4)	455	(7.6)	<0.001
Female	5319	(91.4)	500	(8.6)		5455	(94.7)	303	(5.3)	
Twins	278	(93.3)	20	(6.7)	0.137	259	(90.6)	27	(9.4)	0.036
Family’s socioeconomic status at the time of birth										
Professional	2498	(90.5)	261	(9.5)	0.001	2594	(94.8)	141	(5.2)	<0.001
Skilled workers	3589	(90.9)	358	(9.1)		3644	(93.3)	263	(6.7)	
Unskilled workers	2467	(90.5)	259	(9.5)		2476	(91.9)	218	(8.1)	
Farmers	2061	(92.4)	170	(7.6)		2091	(95.5)	98	(4.5)	
No occupation	131	(82.9)	27	(17.1)		127	(84.1)	24	(15.9)	
Maternal education at the time of birth										
Primary school/no education	7160	(91.3)	680	(8.7)	0.081	7186	(93.0)	543	(7.0)	<0.001
Vocational or secondary school	3016	(90.6)	312	(9.4)		3119	(94.6)	179	(5.4)	
Matriculation or more	480	(88.7)	61	(11.3)		523	(97.4)	14	(2.6)	
Family’s living area at the time of birth										
Town	3605	(91.3)	343	(8.7)	0.203	3605	(92.0)	315	(8.0)	<0.001
Village or other rural area	7216	(90.6)	749	(9.4)		7405	(94.4)	443	(5.6)	

	31-year follow-up					46-year follow-up				
	Participation		No participation			Participation		No participation		
	*n*	(%)	*n*	(%)	*P*-value	*n*	(%)	*n*	(%)	*P*-value
Questionnaire										
Gender										
Male	4167	(71.2)	1688	(28.8)	<0.001	3298	(63.3)	1910	(36.7)	<0.001
Female	4523	(79.5)	1165	(20.5)		3848	(75.1)	1275	(24.9)	
Twins	199	(70.8)	82	(29.2)	0.079	156	(63.9)	88	(36.1)	0.073
Employment (1995/2012)										
Working	6785	(79.7)	1729	(20.3)	<0.001	6602	(72.1)	2551	(27.9)	<0.001
Unemployed	1558	(71.6)	618	(28.4)		544	(46.2)	634	(53.8)	
Socioeconomic status (1995/2012)										
Professional	3690	(82.8)	763	(17.2)	<0.001	4599	(76.6)	1405	(23.4)	<0.001
Skilled workers	1861	(77.3)	540	(22.7)		1346	(62.3)	813	(37.7)	
Unskilled workers	2553	(72.2)	969	(27.8)		1013	(53.7)	874	(46.3)	
Farmers	239	(81.3)	55	(18.7)		169	(71.9)	66	(28.1)	
Education (1997/2014)										
Basic or less	1183	(54.2)	998	(45.8)	<0.001	417	(45.7)	496	(54.3)	<0.001
Secondary	5177	(79.3)	1352	(20.7)		3213	(65.8)	1667	(34.2)	
Tertiary	2315	(84.9)	413	(15.1)		3516	(77.5)	1022	(22.5)	
Marital status (1997/2012)										
Married or in a registered relationship	4057	(82.1)	885	(17.9)	<0.001	4253	(74.3)	1471	(25.7)	<0.001
Other	4633	(70.2)	1968	(29.8)		2893	(62.8)	1714	(37.2)	
Offspring (1997/2012)										
One or more children	4954	(79.4)	1289	(20.6)	<0.001	5774	(71.6)	2290	(28.4)	<0.001
No children	3736	(70.5)	1564	(29.5)		1372	(60.5)	895	(39.5)	
Clinical examination										
Gender										
Male	2880	(65.3)	1530	(34.7)	<0.001	2569	(49.3)	2639	(50.7)	<0.001
Female	3127	(76.4)	966	(23.6)		3263	(63.7)	1860	(36.3)	
Twins	130	(64.4)	72	(35.6)	0.047	122	(50.0)	122	(50.0)	0.040
Employment (1995/2012)										
Working	4850	(73.0)	1798	(27.0)	<0.001	5443	(59.5)	3710	(40.5)	<0.001
Unemployed	1146	(62.6)	684	(37.4)		389	(33.0)	789	(67.0)	
Socioeconomic status (1995/2012)										
Professional	2636	(77.3)	774	(22.7)	<0.001	3886	(64.7)	2118	(35.3)	<0.001
Skilled workers	1331	(69.3)	590	(30.7)		1024	(47.4)	1135	(52.6)	
Unskilled workers	1821	(63.4)	1049	(36.6)		771	(40.9)	1116	(59.1)	
Farmers	208	(75.1)	69	(24.9)		140	(59.6)	95	(40.4)	
Education (1997/2014)										
Basic or less	587	(52.6)	528	(47.4)	<0.001	297	(32.5)	616	(67.5)	<0.001
Secondary	3855	(72.1)	1489	(27.9)		2546	(52.2)	2334	(47.8)	
Tertiary	1563	(78.5)	429	(21.5)		2989	(65.9)	1549	(34.1)	
Marital status (1997/2012)										
Married or in a registered relationship	2847	(76.6)	868	(23.4)	<0.001	3550	(62.0)	2174	(38.0)	<0.001
Other	3160	(66.0)	1628	(34.0)		2282	(49.5)	2325	(50.5)	
Offspring (1997/2012)										
One or more children	3543	(74.3)	1225	(25.7)	<0.001	4755	(59.0)	3309	(41.0)	<0.001
No children	2464	(66.0)	1271	(34.0)		1077	(47.5)	1190	(52.5)	

a Total number of no participation in 1-year follow-up differs from previously published results; the exact information about the target population was no longer available and it was re-selected for these analyses (including individuals who were alive at their 1-year birthday). Originally, the target population was stated as 11 870, resulting in 1049 non-participating children. Here, the target population was estimated as 11 913, resulting in 1092 non-participating children.

In the follow-ups conducted when the cohort members were children, the participation rate was excellent. A total of 10 821 (91.2%) children participated in the 1-year follow-up. The participation was somewhat higher among girls than among boys and differed according to the family’s SES: those with no occupation participated less. The number of participants in the 14-year follow-up was 11 010 (93.6%). Again, girls participated more than boys and the participation differed according to family’s SES (those with no occupation participating less and those with higher SES and farmers participating more). Those who lived in rural areas participated more than those who lived in towns, and children of higher-educated mothers participated more than children of lower-educated mothers.

In the follow-ups that took place in adulthood, the participation rate was bit lower. A total of 8690 (75.3%) cohort members of the target population answered the 31-year postal questionnaire: women answered the postal questionnaire more often than men, the participants were less often unemployed, they were more often from higher social class and with higher education. The participants were also more often married and had children. In all, 6007 (70.7%) cohort members participated in the 31-year clinical examination. Similarly, women were more likely to participate, they were more rarely unemployed, more often from higher social class or farmers and with higher education. Again, they were more often married and had children. More detailed attrition analyses of the 31-year follow-up regarding non-participation among cohort members with psychiatric disorders have been reported earlier.^7^

In the 46-year follow-up, 7146 cohort members (69.2%) answered the postal questionnaires. The participation rates in the four different questionnaires were 66.2%, 65.2%, 55.9% and 54.7%, respectively. Overall, women participated more often than men. The participants were more often employed, married and with children. Cohort members with higher SES and higher education participated more often than the others. In the 46-year clinical examination, the participation rate was 56.5% (*n *= 5832). Again, the participants were more often females, employed and from higher social class. They were also more likely married and to have children and higher education.

## What has it found? Key findings and publications

The Northern Finland Birth Cohort 1966 has been analysed by researchers from a wide range of medical, biological, social and environmental disciplines. There have been many important findings with substantial impact which have been derived directly from the analysis of the NFBC data, resulting in over 1500 peer-reviewed articles or reports. A detailed list of all publications can be found on the NFBC webpage [http://www.oulu.fi/nfbc/publications]. We present here some of the key findings emphasizing the diverse and longitudinal characteristics of the cohort.

As one of the most influential early findings, NFBC1966 established for the first time in a prospective study the association between maternal smoking in pregnancy and the risk of low birthweight.^2^ With later follow-up data, an association between a wide range of early growth measures and blood pressure (BP) at the age of 31 was shown, supporting the role of fetal environment and early life events in the aetiology of BP.^8^ The NFBC1966 early life data remain an invaluable reservoir for linking early life exposures to later health, as demonstrated through recent evidence in a meta-analysis suggesting that DNA methylation may represent a biological mechanism through which exposure to maternal smoking is associated with cardiometabolic risk factors and psychiatric morbidity later in life.^9^ Early life trends in adiposity [body mass index (BMI) and age at BMI peak and rebound) have also been associated with cardiovascular autonomic function in mid-life.^10^

The latest follow-up study with extensive clinical data at 46 years of age has resulted in novel findings showing, for example, that low-grade inflammation is associated with several skin diseases such as atopic eczema and onychomycosis.^11^ It has also been shown that there is an association between metabolic syndrome and periodontal disease, as indicated by deepened periodontal pockets and bone loss.^12^ In addition, with objectively measured physical activity (PA) four distinct clusters were recognized regarding daily temporal patterns of PA and risk for cardiovascular disease.^13^

The NFBC data have enabled new openings in advancing methodology in life course modelling. This is exemplified by the pioneering combination of genetic information with growth modelling analysis.^14^ This modelling identified a subgroup at very high risk of becoming overweight, to be considered in public health settings dealing with obesity prevention. A meta-analysis including the NFBC1966 unravelled for the first time the stratification of early body mass index (BMI) development according to *FTO* genotypes.^15^ Methodological development has also been performed in systems epidemiology with collected biological samples such as a urine nuclear magnetic resonance (NMR) metabolomics platform developed for large-scale studies.^16^

Extensive research in NFBC1966 has been performed on psychiatric diseases and mental health, especially in schizophrenia. A systematic review of 77 original schizophrenia research articles conducted in NFBC1966 before September 2014 was published few years ago.^17^ These studies showed several risk factors associated with schizophrenia such as unwanted pregnancy,^18^ childhood central nervous system viral infections,^19^ familial risk for psychosis^20^ and an antenatally depressed mother.^20^ Other psychiatric studies in NFBC1966 have, for example, reported depression associating with obesity,^21^ insulin resistance,^22^ atopy^23^ and C-reactive protein levels.^24^ A recent study showed generalized anxiety disorder and somatic symptoms associating with frequent health care use.^25^

Research with the longitudinal NFBC1966 data from young adulthood to middle age supports the evidence for a strong association between health and functioning in the society and for the complex biopsychosocial determinants of health. For example, long-term and emerging obesity in mid-adulthood was shown to increase the risk of poor work ability,^26^ and impaired glucose metabolism was associated with diminished participation in working life.^27^ On the other hand, long-term unemployment may predispose to type 2 diabetes.^28^ In the European Union (EU)-funded project, DynaHEALTH, biopsychosocial predictors of blood glucose in middle age were studied with a systematic data-driven approach, providing methodology for studying complex origins of diseases with a variety of biological and psychosocial factors.^29^

## What are the main strengths and weaknesses?

The main strength of NFBC1966 is in its prospective setting and good participation rates (above 50% after 46 years of follow-up). It is a large, population-based birth cohort that comprised nearly 97% (12 231) of all children born in northern Finland in 1966, already with systematically collected high-quality follow-up data since the pregnancy. Over the years, the study protocol has extended from its perinatal and paediatric roots to cover a wide selection of topics on life course health and well-being, to address timely international and regional scientific interests. In particular, the wide variety of biological samples, taken at two different time points 15 years apart, provides an exceptional setting for longitudinal study designs combining early life measurements with adult clinical data. The extensive Finnish national registers and possibility to link information via individual social security number offer important additional sources for multidisciplinary research to address major issues of public health such as metabolic, psychiatric and musculoskeletal problems and multimorbidity. A limitation is, however, that register data have originally been collected for other than research purposes and might not always be optimal for all research questions.

Despite the very high participation rates in the early years of the study, the study has had to face the trend of declining participation rates and loss of follow-up, as in many studies. Still, the participation rates in the latest follow-up study at 46 years of age were up to 69% for the questionnaires and 57% for the clinical examination, highly comparable with the participation rates in cross-sectional European health examination surveys.^30^ There are, however, differences between participants and non-participants (Table 2) in each follow-up, which must be taken into account in analyses.

## Can I get hold of the data? Where can I find out more?

A detailed description of the NFBC1966 data collection is available at [https://www.oulu.fi/nfbc/].^5^ General information is provided about each individual follow-up study together with the questionnaire forms used (in English when possible) and a list of variables formed. Permission to use the data for research purposes can be applied for via electronic material request portal. In the use of data, we follow the EU general data protection regulation (679/2016) and Finnish Data Protection Act (1050/2018). The use of data is based on cohort participants’ written informed consent at their latest follow-up study, which may cause limitations to its use. Different research areas are described on the cohort web page together with a list of contact information. Opportunities for collaboration are welcomed.

## Supplementary Data

Supplementary data are available at *IJE* online.

## Funding

The NFBC1966 follow-up studies were supported by the University of Oulu (Grants no. 65354, 24000692), Oulu University Hospital (Grants no. 2/97, 8/97, 24301140), National research funding via City of Oulu, Ministry of Health and Social Affairs (Grants no. 23/251/97, 160/97, 190/97), National Institute for Health and Welfare, Helsinki (Grant no. 54121), Regional Institute of Occupational Health, Oulu, Finland (Grants no. 50621, 54231), and ERDF European Regional Development Fund (Grant no. 539/2010 A31592). The research on NFBC1966 data has been supported in part by the the European Commission research and innovation program Horizon 2020 under the following projects: DynaHealth (Grant no. 633595), LifeCycle (Grant no. 733206), EUCANCONNECT (Grant no. 824989), LongITools (Grant no. 873749), EarlyCause (Grant no. 848158); and the Joint Programming Initiative Healthy Diet Healthy Life (PREcisE project - ZonMw the Netherlands Grant no. P75416).

## Supplementary Material

dyab109_Supplementary_Data
