# Supplementary material for: Cohort Profile: 46 years of follow-up of the Northern Finland Birth Cohort 1966 (NFBC1966)
Source: Int J Epidemiol. 2021 Aug 30;50(6):1786–1787j. doi: 10.1093/ije/dyab109 (PMC8743124; doi:10.1093/ije/dyab109)
Supplement: dyab109_Supplementary_Data [file dyab109_supplementary_data.docx]

**Table S1.** Subpopulation studies in the Northern Finland Birth Cohort 1966 performed in 1965 to 2011.

| **Description of the study/subpopulation** | **Collected data** | **N** | **Begin** | **End** | **Reference** |
| --- | --- | --- | --- | --- | --- |
| Obstetric data of children whose mothers were living in the city of Oulu. | Prenatal information concerning mother's health condition during pregnancy was obtained from the antenatal cards requested from the antenatal clinics. | 1,734 | 1965 | 1967 | No main reference. Data has been used later as part of the 14-year follow-up study. |
| Children who were treated in the newborn nursery during perinatal period. | Data was retrospectively collected from case records in children's departments of central hospitals. | 628 | 1966 | 1967 | Rantakallio P, von Wendt L, Koivu M. Prognosis of perinatal brain damage: a prospective study of a one-year birth cohort of 12 000 children. *Early Hum. Dev* 1987; **15**:75-84. |
| Children with congenital heart defects. | Data concerning cohort's children with congenital heart defects (CHD). | 352 | 1967 | 1967 | [Nuutinen M, Koivu M, Rantakallio P. Long-term outcome for children with congenital heart defects. A study from 1-year birth cohort born in 1966 in northern Finland.](http://www.ncbi.nlm.nih.gov/pubmed/2590316) *[Arctic Med Res](http://www.ncbi.nlm.nih.gov/pubmed/2590316)* [1989;](http://www.ncbi.nlm.nih.gov/pubmed/2590316) **[48](http://www.ncbi.nlm.nih.gov/pubmed/2590316)**[:175-84.](http://www.ncbi.nlm.nih.gov/pubmed/2590316) |
| Neurological examination at 1 year of age. | Neurological status of children considered to be at risk based on their perinatal history. | 722 | 1967 | 1967 | No main reference. Data has been used when identifying neurological cases. |
| Twin study | Data collected of twins and controls. | 652 | 1966 | 1985 | Moilanen I, Rantakallio P. The growth, development and education of Finnish twins: a longitudinal follow-up study in a birth cohort from pregnancy to nineteen years of age. *Growth Dev Aging* 1989; **53**:145-50. |
| Neurological illnesses and abnormalities up to the age of 5 years. | Detailed information was obtained from hospital records in Oulu and Lapland districts on children who had visited hospital because of a neurological disease. | 295 | 1966 | 1971 | von Wendt L, Rantakallio P, Saukkonen A-L, Tuisku M, Mäkinen H. Cerebral palsy and additional handicaps in a 1-year birth cohort from Northern Finland - a prospective follow-up study to the age of 14 years. *Ann Clin Res* 1985; **17**:156-161.  von Wendt L, Rantakallio P, Saukkonen A-L, Mäkinen H. Epilepsy and associated handicaps in a 1-year birth cohort in Northern Finland. *Eur J Pediatr* 1985; **144**: 149-151. |
| Ophthalmological data up to the age of 5 years. | Data concerning visits to the ophthalmological departments and clinics of central hospitals of the cohort-district. | 632 | 1966 | 1971 | Rantakallio P, Krause U, Krause K. The use of the ophthalmological services during the preschool age, ocular findings and family background. *J Pediatr Ophthal Strab*. 1978; **15**:253-258.  Krause K, Krause U, Rantakallio P. Regional differences in the use of ophthalmological services during the pre-school period. *Nordic Council Arct Med Res Rep* 1978; **23**:20-25. |
| Central nervous system (CNS) infections up to 14 years of age. | Data on CNS infections recorded during 1966 -1980. | 174 | 1980 | 1980 | von Wendt L, Rantakallio P. Congenital malformations of the central nervous system in a 1-year birth cohort followed to the age of 14 years. *Childs Nerv Syst* 1986;**2**:80-2. |
| Cerebral palsy, a subpopulation from the 14-year follow-up study. | The data were collected during 14-year follow-up, determining all the neurological diseases by examining epicrises from neurology policlinics and hospital discharge records, and registers of the Social Insurance Institution of Finland. | 69 | 1980 | 1981 | von Wendt L, Rantakallio P, Saukkonen AL, Tuisku M, Mäkinen H. Cerebral palsy and additional handicaps in a 1-year birth cohort from northern Finland — a prospective follow-up study to the age of 14 years. *Ann Clin Res* 1985;**17**:156-61. |
| Epilepsy up to age 14 years. | The data were collected during 14-year follow-up by examining epicrises from neurology policlinics and hospital discharge records, and registers of the Social Insurance Institution of Finland. | 208 | 1980 | 1981 | von Wendt L, Rantakallio P, Saukkonen A-L, Mäkinen H. Epilepsy and associated handicaps in a 1-year birth cohort in Northern Finland. *Eur J Pediatr* 1985; **144**: 149-151. |
| Delays in school performance up to the age of 14 years. | Follow-up data on the mental and physical development of the children were collected at various ages; the last follow-up was in 1980 and 1981, when the children were 14 years old. | 176 | 1980 | 1981 | Partly used in Rantakallio P, von Wendt L. Mental retardation and subnormality in a birth cohort of 12,000 children in Northern Finland. *Am J Ment Defic* 1986; *90*:380-387. |
| Intellectually disabled | Data of intellectually disabled (IQ below 85), recorded at the age of 14-years. | 326 | 1980 | 1981 | Rantakallio P, von Wendt L. Mental retardation and subnormality in a birth cohort of 12,000 children in Northern Finland. *Am J Ment Defic* 1986; **90**:380-387. |
| Cohort members with registered visits to ophthalmologist or the Oulu University Hospital Eye Clinic. | Ophthalmological records of the cohort children were collected from the offices of 23 private ophthalmologists and from the Oulu University Hospital. | 1,939 | 1980 | 1981 | Krause U, Krause K, Rantakallio P. Sex differences in refraction errors up to the age of 15. *Acta Ophthalmol* 1982; **60**:917-926. |
| School-study on unwanted and wanted persons at the age of 16. | A series of 231 unwanted children, 123 boys and 108 girls, and 227 controls (124 boys and 103 girls). Teachers in Finland and in Sweden were asked to fill in a questionnaire. | 559 | 1980 | 1982 | Myhrman A. Family relation and social competence of children unwanted at birth. A follow-up study at the age of 16. *Acta Psychiatr Scand* 1988; **77**:181-187. |
| Growing-up study 1983 | Persons (unwanted and controls in Finland and Sweden) at 16 years old interviewed by Antero Myhrman. | 177 | 1983 | 1983 | Myhrman A. Family relation and social competence of children unwanted at birth. A follow-up study at the age of 16. *Acta Psychiatr Scand* 1988; **77**:181-187. |
| Intelligence quotient (IQ) up to 14 years. | IQ- tests for those with lower than normal school performance. | 495 | 1983 | 1983 | Rantakallio P, von Wendt L. Mental retardation and subnormality in a birth cohort of 12,000 children in Northern Finland. *Am J Ment Defic* 1986; **90**:380-387. |
| Hearing at age of 14. | Hearing status was studied at the age of 14 by health questionnaire. Audiometry results were obtained from school health care. | 1,372 | 1983 | 1983 | Sorri M, Rantakallio P. Prevalence of hearing loss at the age of 15 in a birth cohort of 12 000 children from Northern Finland. *Scand Audiol* 1985; **14**:203-207. |
| Neurological handicaps | Neurological data of children from the age of five until the age of 14 years from neurological outpatient clinics. | 847 | 1983 | 1983 | Data has been collected by Lennart von Wendt and it has been used since in several publications. |
| Follow-up study of the ophthalmological data collected at the age of 14; Myopia study at the age of 20 years. | Data concerning cohort members who had myopia in the ophthalmological study at the age of 14 and their controls. | 502 | 1986 | 1986 | Krause U, Rantakallio P, Koiranen M, Möttönen J. The development of myopia up to the age of twenty and a comparison of refraction in parents and children. *Arctic Medical Research* 1993; **52**:161-165.  Möttönen J, Oja H, Krause U, Rantakallio P. Application of random coefficient regression model to myopia data: A case study. *Biometrical Journal* 1995; **37/6**:657-672 |
| Health and life satisfaction of male cohort members at 1990. | Questionnaire on health and life habits to a random subsample of males in the cohort at the age of 24 years. | 2,500 | 1990 | 1990 | Pietilä A-M. Elämänhallinta ja terveys. Pitkittäistutkimus pohjoissuomalaisilla nuorilla miehillä. /Coping and health. Longitudinal study on men from northern Finland. *Acta Univ Oul Medica* D 313, 1994 (Thesis, In Finnish) |
| Psychiatric substudy of NFBC1966 follow-up study at 31 years. | Participants living in the city of Oulu were invited for a two-phase psychiatric interview study. | 1,310 | 1997 | 1998 | Veijola J, Jokelainen J, Läksy K, Kantojärvi L, Kokkonen P, Järvelin MR, Joukamaa M. The Hopkins Symptom Checklist-25 in screening DSM-III-R axis-I disorders. *Nord J Psychiatry* 2003;57(2):119-23. |
| Follow-up study of individuals with psychosis and their controls. | Psychiatric interviews, questionnaires, brain MRI scans, cognitive tests. | 191 | 1999 | 2001 | Haapea M, Miettunen J, Veijola J, Lauronen E, Tanskanen P, Isohanni M. Non-participation may bias the results of a psychiatric survey. An analysis from the survey including magnetic resonance imaging within the Northern Finland 1966 Birth Cohort. *Soc Psychiatry Psychiatr Epidemiol* 2007; **42**:403-9. |
| Association of androgenetic alopecia with endocrine and metabolic parameters in the NFBC1966. | A postal questionnaire and an evaluation form regarding loss of hair for all men in NFBC1966. | 3,128 | 2005 | 2005 | Data has not been published. |
| Follow-up study of individuals with psychosis, their controls and siblings. Includes new participants, but also individuals who participated to an earlier follow-up in 1999-2001. | Psychiatric interviews, questionnaires, brain MRI scans, cognitive tests. | 312 | 2008 | 2011 | Jääskeläinen E, Haapea M, Rautio N, Juola P, Penttilä M, Nordström T, Rissanen I, Husa A, Keskinen E, Marttila R, Filatova S, Paaso TM, Koivukangas J, Moilanen K, Isohanni M, Miettunen J. Twenty Years of Schizophrenia Research in the Northern Finland Birth Cohort 1966: A Systematic Review. *Schizophr Res Treatment* 2015; 524875. |
| Ophthalmological data was collected at 46 – 48 years. | Clinical eye examination (visus, refraction, intraocular pressure, thickness of cornea, field of vision, eye photographing). | 3,070 | 2012 | 2014 | Saarela V, Karvonen E, Stoor K, Hägg P, Luodonpää M, Kuoppala J, Taanila A, Tuulonen A. The Northern Finland Birth Cohort Eye Study: Design and baseline characteristics. *BMC Ophthalmol* 2013; **13:**51. |
